# Vitamin D Imbalance and Hydro-Electrolyte Disturbances in Hospitalized Children: A Comparation Between Post-COVID-19 Status and SARS-CoV-2/EBV Coinfection

**DOI:** 10.3390/biomedicines13051233

**Published:** 2025-05-19

**Authors:** Carmen Loredana Petrea (Cliveți), Diana-Andreea Ciortea, Gabriela Gurău, Nicoleta Mădălina Matei, Ciprian Adrian Dinu, Simona-Elena Bergheș (Oprea), Gabriela Isabela Verga (Răuță), Sorin Ion Berbece

**Affiliations:** 1 Faculty of Medicine and Pharmacy, University “Dunarea de Jos” of Galati, 800008 Galati, Romania; carmen.petrea@ugal.ro (C.L.P.); madalina.matei@ugal.ro (N.M.M.); c.dinu@ugal.ro (C.A.D.); eo111@student.ugal.ro (S.-E.B.); gabriela.verga@ugal.ro (G.I.V.); sorin.berbece@ugal.ro (S.I.B.); 2 Emergency Clinical Hospital for Children “Sf. Ioan”, 800487 Galati, Romania; 3 Emergency Clinical Hospital for Children “Maria Sklodowska Curie”, 041451 Bucharest, Romania

**Keywords:** post-COVID-19 infection status, SARS-CoV-2/EBV coinfection, children, 25(OH)D, hypovitaminosis D, hypervitaminosis D, hydro-electrolyte disturbances

## Abstract

**Background/Objectives:** SARS-CoV-2 infection has the potential to cause multi-organ involvement and, when associated with Epstein–Barr virus (EBV) coinfection, may worsen the course of disease in pediatric patients by influencing the immune response. **Methods:** Our retrospective–observational study included 406 hospitalized children with post-COVID-19 status or SARS-CoV-2/EBV coinfection. **Results:** Hypovitaminosis D was more common in the coinfected sublot (59.18%) than in the COVID-19 one (50.74%), with a higher frequency of severe vitamin D deficiency (16.33% vs. 7.35%). Hypovitaminosis D was significantly associated with female sex (*p* = 0.033) only in the COVID-19 subgroup. Hypervitaminosis D, although rare, was only associated with severe forms of the disease (7.69%). Between clinical severity and vitamin D level, a statistically significant association of moderate intensity was identified only in the COVID-19 subgroup (χ^2^ = 11.708, φ = 0.293, *p* = 0.020). In the same subgroup, a significant correlation was found between vitamin D levels and serum potassium values (χ^2^ = 10.527, *p* = 0.032). Moreover, in the COVID-19 subgroup, an association between abnormal sodium levels and increased D-dimer levels was found (χ^2^ = 7.074, *p* = 0.029). **Conclusions:** These results underline the importance of monitoring immunologic, vitamin, and electrolyte imbalance in the management of these cases and highlight the need for personalized therapeutic strategies to prevent long-term complications.

## 1. Introduction

The Coronavirus Disease 2019 (COVID-19), officially declared a pandemic by the World Health Organization (WHO) on 11 March 2020 [1], has had a significant impact on global health, and the effects on children have become an important topic of research. During this period, an increase in pediatric cases of SARS-CoV-2 infection has been observed [2,3], primarily presenting as mild or asymptomatic forms with an extremely low mortality rate [4,5]. There is increasing evidence to suggest that even mild forms of the disease may cause post-viral complications, especially in the context of immunologic and electrolyte imbalances that can occur after COVID-19 infection [6].

Studies on the late effects after COVID-19 infection are still in their early stages, especially in the pediatric population, where investigations on hydro-electrolyte disturbances and vitamin D remain limited. SARS-CoV-2 infection may also trigger multisystem inflammatory syndrome and may influence the function of several organs, including electrolyte homeostasis. These dysregulations may be amplified in the presence of viral coinfections, such as Epstein–Barr virus (EBV) [7,8,9], an agent with lymphocytic tropism and reactivation potential, recognized for its contribution to persistent immune dysfunction [10,11,12].

SARS-CoV-2/EBV coinfection is considered a potentially aggravating factor, mainly because it modulates the cellular immune response and favors a persistent inflammatory profile. Proposed mechanisms include EBV reactivation amid SARS-CoV-2-induced immunosuppression, altered ACE2 receptor expression, and decreased CD8+ lymphocyte counts [13,14]. This immune dysfunctional context may influence not only the clinical course but also the biochemical and electrolyte profile of pediatric patients.

In this complex landscape, vitamin D, often called the “sunshine vitamin”, has attracted increasing interest due to its pleiotropic effects in supporting the immune system and regulating inflammatory responses. Vitamin D is synthesized endogenously by the action of UVB radiation on the skin, but it requires two essential activation (hydroxylation) steps: hepatic (25-hydroxylation) and renal (1α-hydroxylation), with the circulating 25(OH)D form being considered the most reliable indicator of vitamin status [15,16].The presence of vitamin D receptors (VDRs) in most immune cells supports its involvement in the regulation of both innate and adaptive immunity [17]. Several studies have emphasized the role of calcitriol, a vitamin D agonist, in protecting against acute lung injury by modulating the renin–angiotensin system [18]. Consequently, the link between low vitamin D levels and the increased risk of respiratory infections or severe complications in patients infected with SARS-CoV-2 [19], has been demonstrated. At the other end of the spectrum, optimal vitamin D levels could contribute to the reduction of inflammatory reactions mediated by interleukin-6 (IL-6) and interferon gamma (IFNγ), which are predictors of a poor prognosis in severe cases of COVID-19 [20]. In addition, recent research has suggested that the rate of viral replication can be diminished by the action of cathelicidins and defensives, which are induced by vitamin D [21].

At the pediatric level, the data are less conclusive. Existing studies, mainly focused on adult patients, have provided contradictory results regarding the association between vitamin D levels and the severity of COVID-19 infection [22,23]. Some research has observed negative correlations between 25(OH)D levels and inflammatory markers, such as C-reactive protein (CRP) or neutrophil-to-lymphocyte ratio (NRL). In contrast, others have shown no significant links [24,25,26,27]. Moreover, studies highlighting the existence of a correlation between vitamin D status and its impact on the evolution after SARS-CoV-2 infection or associated coinfections, are minimal. Additionally, hypervitaminosis, although rarely reported, can have significant adverse effects, including hypercalcemia, nephrocalcinosis, and metabolic imbalances. The role of vitamin D in the context of COVID-19 remains insufficiently explored.

On the other hand, hydro-electrolyte disorders, such as hyponatremia or hypokalemia, have been described especially among adult patients with severe forms of COVID-19, being correlated with an increased risk of mortality and morbidity [28].

In a pediatric context, research on the link between vitamin status, electrolyte balance, and biomarkers of inflammation is extremely limited, not only in the context of active SARS-CoV-2 infection, but especially in cases of associated coinfections or in the post-COVID-19 infection state. Studies have highlighted the importance of the exaggerated post-infectious immune response induced by SARS-CoV-2, which leads to the release of a large amount of proinflammatory cytokines, a phenomenon known as the “cytokine storm”. Interleukin-1β and IL-6 are thought to stimulate the secretion of arginine vasopressin, which can lead to the onset of inadequate antidiuretic hormone secretion syndrome (SIADH) and, implicitly, hyponatremia in the context of SARS-CoV-2 infection. Before the COVID-19 vaccine era, hydro-electrolyte disorders were commonly observed, especially in critically ill adult patients [29,30].

Thus, it becomes essential to explore how vitamin D imbalances and hydro-electrolyte disorders may reflect the severity and post-infectious course among children affected by COVID-19, with or without EBV coinfection. Therefore, identifying potential risk factors, such as vitamin D deficiency or excess, as well as electrolyte abnormalities associated with post-COVID-19 infection or EBV coinfection, can bring value in risk stratification and the development of personalized therapeutic strategies.

The present study aims to evaluate vitamin D status and electrolyte balance in children hospitalized secondary to a COVID-19 infection, compared to those with SARS-CoV-2/EBV coinfection, providing new insights into the immunological and metabolic implications of these viral infections in the pediatric population.

## 2. Materials and Methods

For this study, we performed a retrospective analysis of patients admitted to the St. Ioan Children’s Emergency Hospital in Galati, Romania, between 1 December 2022 and 30 November 2024. The patients were diagnosed with post-COVID-19 infection status or SARS-CoV-2/EBV coinfection.

Patients with positive immunoglobulin G (IgG) antibodies for SARS-CoV-2 or Epstein–Barr virus (EBV), considered to have had previous infections with these viruses, were included in this study, provided there was no indication of active disease at the time of testing. In the case of EBV, the term “latent infection” was used to denote the presence of the virus in an inactive form without acute symptoms. Epstein–Barr virus reactivations were investigated in the context of persistent clinical symptoms and elevated inflammatory markers.

A total of 406 children and adolescents were included in the study, divided into two groups based on their immunological status. The first group consisted of 285 patients with a post-COVID-19 infection status, and the second group comprised 121 patients diagnosed with a previous SARS-CoV-2/EBV coinfection. The post-COVID-19 infection status was determined based on an IgG antibody titter to SARS-CoV-2 that was outside the reference range, indicating a post-infectious immune response. SARS-CoV-2/EBV coinfection was confirmed by the simultaneous identification of specific antibodies to both viruses, including anti-EBV (VCA) and anti-EBV (EBNA) IgG and IgM, as well as anti-SARS-CoV-2 IgG and IgM, providing clear serologic evidence of the concomitant presence of the two infections (Table 1).

The collected data included information on the period and duration of hospitalization, fluctuations in inflammatory markers, electrolyte balance, and vitamin D levels.

In analyzing the relevant biological parameters, we established the appropriate reference ranges, which were tailored explicitly by age category according to pediatric practice (Table 1). We also provided details on the analytical methods and equipment used in their assessment.

To assess vitamin D status, the serum concentration of 25-hydroxyvitamin D (25-OH vitamin D) was analyzed. This form includes cholecalciferol (vitamin D_3_) and ergocalciferol (vitamin D_2_). 25-hydroxyvitamin D, with a prolonged half-life of 2–3 weeks, is the primary circulating reservoir and the best indicator of the overall vitamin D status [31]. More than 95% of 25-OH vitamin D dosage in serum is represented by 25-OH vitamin D; measurable levels of 25-OH vitamin D_2_ in serum were found only in patients taking medication containing vitamin D_2_ [32]. The test used in the hospital clinical laboratory and applied in our research determines 25-OH total vitamin D, its variations, and those outside the reference range, indicating the rate of vitamin D according to the duration and intensity of exposure to sunlight, diet, or nutritional supplements used.

Analysis of anti-SARS-CoV-2 IgG and IgM (immunoglobulin G and M) antibodies, procalcitonin and 25-OH vitamin D, was performed using the chemiluminescence method on the YHLO-IFLASH 1800 system (YHLO Biotech Co., Ltd., Shenzhen, China). Detection of anti-EBV IgG (VCA), anti-EBV IgM (VCA), and anti-EBV IgG (EBNA) antibodies was performed on the Gemini apparatus, using the enzyme-linked immunosorbent assay (ELISA) method.

The concentrations of serum sodium (Na) and serum potassium (K) electrolytes were determined by the potentiometric method using the Vitros 4600 Chemistry System Chemistry Analyzers (Ortho Clinical Diagnostics, Inc., Rochester, NY, USA).

Changes in serum sodium and potassium concentrations that exceeded the reference range limits, reported according to age and sex, were considered electrolyte disturbances. Hypovitaminosis D was defined as a vitamin D (25-OH vitamin D) value lower than 30 ng/dL, while hypervitaminosis D was described as a vitamin D value higher than 50 ng/dL.

This study was approved by the Medical Council of the St. Ioan Children’s Emergency Clinical Hospital Galati, as per the meeting minutes dated 30 January 2025, document number C1972.

### 2.1. Study Variables

Demographic data, including age, sex, and duration of hospitalization, as well as biomarker results such as serum electrolytes, acute phase reactants, and 25-OH vitamin D, were extracted from the medical records of the pediatric patients. Data collection was performed by the ethical standards of institutional research committees and/or the Declaration of Helsinki (revised 2013).

Descriptive and inferential statistical methods, implemented by IBM SPSS Statistics 27, were used to analyze the characteristics of the samples and the relationships between variables. The data were initially collected and organized using Microsoft Excel (Office 365), and the results were presented as text, graphs, and tables. Descriptive analysis was performed using indicators of central tendency, such as the mean and media, as well as measures of dispersion (standard deviation). The distribution of continuous variables was assessed visually by histograms and box plots, as well as normality tests, including the Shapiro–Wilk test.

For the inferential analysis, non-parametric methods were used, appropriate to the type of variables and the hypotheses of the tests. The comparison of two independent groups was performed by the Mann–Whitney U (U) test, applied to ordinary or continuous variables without normal distributions. Differences between two independent groups were assessed by the Kruskal–Wallis H test, used in the absence of normality or homogeneity of dispersions. The association between nominal or dichotomous variables was analyzed with the Chi-square test, and the strength of the association between variables was quantified by the correlation coefficients Phi (φ) and Cramer’s Phi (φ), and V. The Phi coefficient ranges from −1 to +1, with the sign indicating the direction of the association and the magnitude reflecting the strength of the relationship. For Cramer’s coefficient V, it ranges between 0 and 1; these indicate the strength of the association.

For numeric or ordinal variables, the Spearmen correlation indicator (rs) was applied, with values ranging from −1 to +1, where 0 indicates the absence of a relationship. This test enabled the identification of the strength and direction of the relationships between the analyzed variables.

The data was guaranteed with a probability of 95% and a maximum permissible error of ±5%. Values of *p* < 0.05 were considered statistically significant.

We used the acronym” COVID” to refer to the cohort of confirmed patients with post-COVID-19 infection status in the tables and graphs, and the abbreviation” COVID-19 + EBV” to denote the cohort with SARS-CoV-2/EBV coinfection.

### 2.2. Inclusion Criteria

To be included in the study, patients had to be children and adolescents under 18 years of age who were confirmed with post-COVID-19 infection status by specific molecular tests or with SARS-CoV-2/EBV coinfection based on serologic tests (positive IgM/IgG antibodies to EBV-VCA, or IgG anti EBV-EBNA). The study was conducted on patients hospitalized in the St. Ioan Children’s Emergency Clinical Hospital in Galati, between 1 December 2022 and 30 November 2024. Regarding the use of data for research purposes, informed consent was obtained from parents or legal representatives of pediatric patients. Only patients with available detailed clinical information, such as clinical manifestations, medical history, and results of paraclinical investigations, were included. For patient selection, vaccination status against COVID-19 was not considered, as vaccination recommendations for children over 5 years of age were issued by the US Centers for Disease Control and Prevention at the end of 2021 [33], and vaccination status was not available in medical records.

### 2.3. Exclusion Criteria

Patients with severe pre-existing comorbidities, such as chronic kidney disease, severe rickets of other etiology, and those with congenital or acquired immunodeficiencies not caused by COVID-19 or EBV, that could have affected hydro-electrolyte or vitamin D metabolism were excluded from the study. Additionally, children and adolescents treated with systemic corticosteroids, immunomodulators, or electrolyte supplements within the 30 days prior to hospitalization were also excluded, as these treatments could have influenced paraclinical results.

## 3. Results

### 3.1. Demographic Data Distribution of Cases in the Two Subgroups

The retrospective analysis included 406 patients confirmed to have anti-SARS-CoV-2 IgG antibodies, of whom 70.20% (*n* = 285) had a post-COVID-19 infection status only and 29.8% (*n* = 121) had a SARS-CoV-2/EBV coinfection.

Patient ages in both subgroups ranged from 0 to 17 years. In the cohort with post-COVID-19 infection status, the mean age was 6.24 years (SD 5.26, CI: [5.62, 6.85]), and the median age was 4 years. In the subgroup with SARS-CoV-2/EBV coinfection, the mean age was 7.5 years (standard deviation 5.30, CI: [6.54, 8.45]), and the median was 7 years.

**Figure 1:**
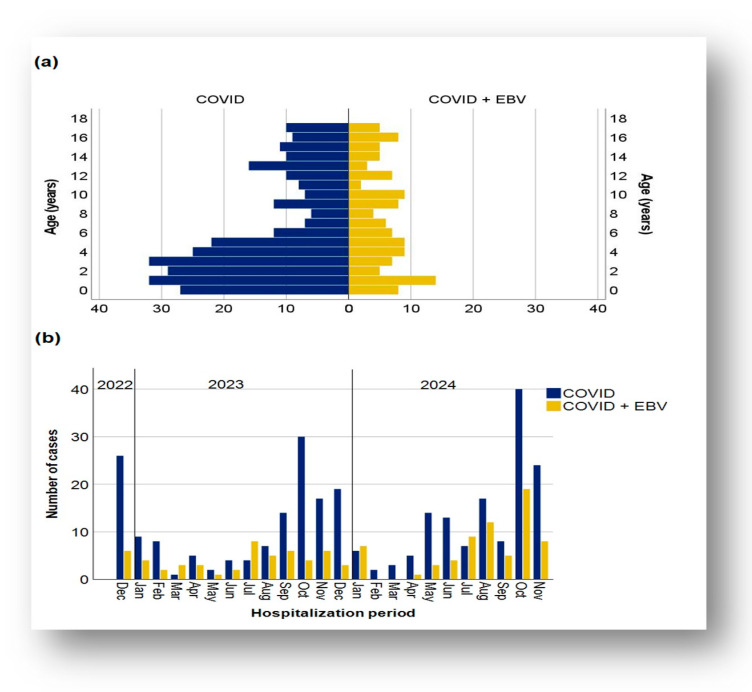
(**a**,**b**) Distribution of pediatric cases in the two subgroups: COVID-19 (*n* = 285, 100%) and COVID-19 + EBV (*n* = 121, 100%) by age group (**a**) and by hospitalization period during the two-year study (**b**). This figure highlights the monthly distribution and variation in case incidence during the study period, offering insights into temporal patterns of hospitalization.

The age group distribution revealed a predominance of cases in the 11–18-years age range in both subgroups, whereas the 1–3-year and 6–11-year age groups were less represented in the COVID-19 + EBV and COVID-19 subgroups, respectively (Figure 1a).

The application of the Mann–Whitney U test revealed statistically significant differences between the age of patients with post-COVID-19 infection status (mean rank, 194.73) and those with SARS-CoV-2/EBV coinfection (mean rank, 224.15): U = 14,744, Z = −2.316, and *p* = 0.021. Thus, the presence of COVID-19 + EBV coinfection was associated with significantly higher ages compared to post-COVID-19 infection status.

Regarding the gender distribution, it was similar and balanced between the two subgroups, with a slight difference in the gender proportion within each subgroup. Male sex was more common in both cases with post-COVID-19 infection status (50.53%) and SARS-CoV-2/EBV coinfection (53.73%).

The distribution of cases by month showed a distinct dynamic in the evolution of cases. In the cohort with post-COVID-19 infection status, the number of cases showed an apparent cyclicity, with peaks in October 2023 and 2024, suggesting a possible association with the seasonality of the phenomenon (Figure 1b). This subgroup also showed significant monthly fluctuations, with considerable variability in the number of cases. Moreover, the epidemic dynamics were marked by increases and decreases throughout the year. In contrast, the subgroup with COVID-19 + EBV coinfection displayed a more variable dynamic of monthly fluctuations, with a peak in October 2024, but no instances of coinfection in February and March 2024. The recorded oscillations suggest significant variability without a uniform trend, and the upward and downward slopes reflect an irregular pattern influenced by contextual factors and the overall evolution of the pandemic.

In total, 7.62% of the cases studied had comorbidities, including bronchial asthma (0.74%); overweight (0.99%); autism spectrum disorders (0.49%); neurovegetative impairment; or autoimmune diseases such as systemic lupus erythematosus (SLE) or type I diabetes. Patients were under appropriate therapeutic management, monitored, and did not have associated complications.

### 3.2. Characteristics of Clinical Evolution: Severity and Duration of Hospitalization

#### 3.2.1. Severity of the Disease

Most patients had moderate forms of the disease, and a small number had severe forms (Table 2). Although there were no significant differences between subgroups, a slightly increased incidence of severe forms was observed among COVID-19 + EBV patients (7.44%) compared to the COVID-19 subgroup (2.46%). Disease severity was assessed based on clinical manifestations correlated with changes in biological markers and duration of hospitalization. Based on these factors, the clinical forms of the disease were defined as mild, moderate, or severe.

##### Inflammatory Markers and Severity of Disease

The presence of a second infectious agent, such as Epstein–Barr (EBV), can significantly influence the body’s inflammatory response. To assess the impact of infection or coinfection on the inflammatory response, several acute-phase reactants, including C-reactive protein (CRP); erythrocyte sedimentation rate (ESR); interleukin-6 (IL-6); procalcitonin; and markers of coagulation activation, such as D-dimer, were analyzed. These markers were correlated with patients’ immunologic status to identify significant differences between subgroups.

C-reactive protein (CRP) is a significant predictor of disease severity, playing an essential role in identifying patients at increased risk of severe disease, both in the subgroup with post-COVID-19 infection status and in the subgroup with SARS-CoV-2/EBV coinfection. Patients with elevated CRP values exhibited more severe clinical manifestations compared to those with normal CRP values. Thus, mild forms predominated among patients with normal CRP (54.10%), whereas moderate forms were more frequent in patients with elevated CRP (61.59%)**.** No significant differences were observed in severe forms (2.46% of cases with normal CRP versus 2.65% with elevated CRP).

The Chi-square test revealed a significant association between increased CRP and severity of manifestations (χ^2^ = 9.326, *p* = 0.009), and the Phi coefficient (φ = 0.185) indicated a weak relationship between the two variables. These results suggested that patients with elevated CRP values were more prone to develop moderate forms of disease. In contrast, cases with normal CRP values had a propensity toward mild forms of disease.

**Figure 2:**
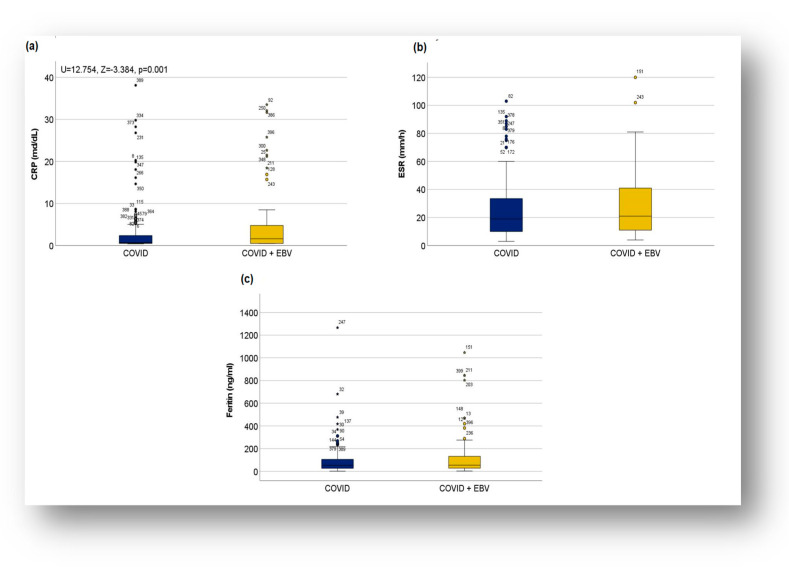
Comparative distribution of inflammatory markers (CRP (**a**), ESR (**b**), and ferritin (**c**)) between the COVID-19 only and COVID-19 + EBV subgroups. The figures illustrate differences in central tendency and variability of acute-phase reactants between the two clinical groups.

Furthermore, in the same analysis, a statistically significant association was observed between CRP and COVID-19 + EBV coinfection (χ^2^ = 5.312, *p* = 0.021), and the Chi-square test indicated a direct, yet weak relationship (φ = 0.117). Additionally, the Mann–Whitney U test showed that patients with coinfection had higher CRP values more frequently compared to the patients with post-COVID-19 infection status, with values within a significantly different range (U = 12.754, Z = −3.384, *p* = 0.001) (Figure 2a).

For the other acute-phase reactants, such as erythrocyte sedimentation rate (ESR), ferritin, and D-dimer, a higher incidence of pathologic values was observed in the COVID-19 + EBV subgroup, without significant differences between subgroups. More than 60% of patients in the COVID-19 subgroup had elevated ESR and D-dimer values, compared to more than 70% in the COVID-19 + EBV subgroup. Ferritin had normal values in most patients in both subgroups (Figure 2c and Table 3). Regarding interleukin-6 (IL-6) and procalcitonin, elevated values were more frequent in the COVID-19 subgroup, where extreme values were also recorded. IL-6, although investigated less frequently, had elevated values in all investigated patients in the COVID-19 subgroup (100%), compared to 50% in the COVID-19 + EBV subgroup. In contrast, procalcitonin had normal values in most cases in both subgroups (77.61% in COVID-19 subgroup, and 83.33% in COVID-19 + EBV subgroup) and less frequently showed elevated values (Table 3). These data suggest an enhanced inflammatory response and coagulation activation in COVID-19 + EBV coinfection.

#### 3.2.2. Length of Hospitalization in the Analyzed Subgroups

**Figure 3:**
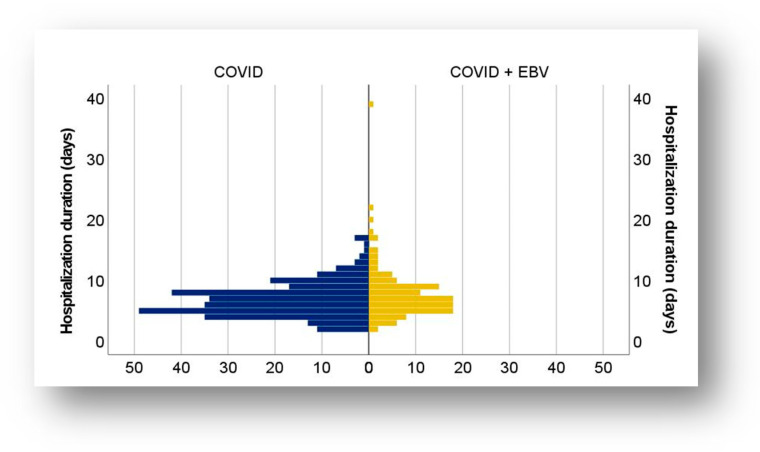
Distribution of hospitalization duration (days) among pediatric patients with COVID-19 and COVID-19 + EBV coinfection. The figure illustrates the variation in length of hospital stay across the two groups. Each bar reflects how many patients experienced a specific number of days of hospitalization.

The duration of hospitalization of the 406 patients ranged from 2 to 39 days in both subgroups, with short hospitalizations of less than one week predominating (Table 2). Almost one-third of the patients required hospitalization between 8 and 14 days, while hospitalizations longer than 14 days were exceptional (Figure 3).

In the COVID-19 subgroup, the mean length of hospitalization was 6.83 days (standard deviation 2.85; CI: [6.50;7.16]) with a median of 6 days. In comparison, in the subgroup with COVID-19 + EBV coinfection, the mean duration was 7.91 days (standard deviation 4.56; CI: [7.09; 8.73]), and the median was 7 days (Table 2). One exceptional case in the COVID-19 + EBV subgroup had an extreme hospitalization duration of 39 days, whereas in the COVID-19 subgroup, the longest hospitalization was 17 days (Figure 3). The minimum duration of hospitalization in both subgroups was 2 days.

According to the statistical analysis using the Mann–Whitney U test, patients with COVID-19 + EBV coinfection had a significantly longer duration of hospitalization than those with post-COVID-19 infection status (U = 15.091, Z = −2.003, *p* = 0.045). Spearman’s correlation coefficient analysis revealed a weak, inverse correlation between patient age and duration of hospitalization in the coinfected subgroup, suggesting that the duration of hospitalization increased slightly as the child’s age decreased (rs = −0.257, *p* = 0.004). In contrast, in the subset with post-COVID-19 infection status, the duration of hospitalization was not influenced by age.

##### Length of Hospitalization in the Context of Severity of Illness

**Figure 4:**
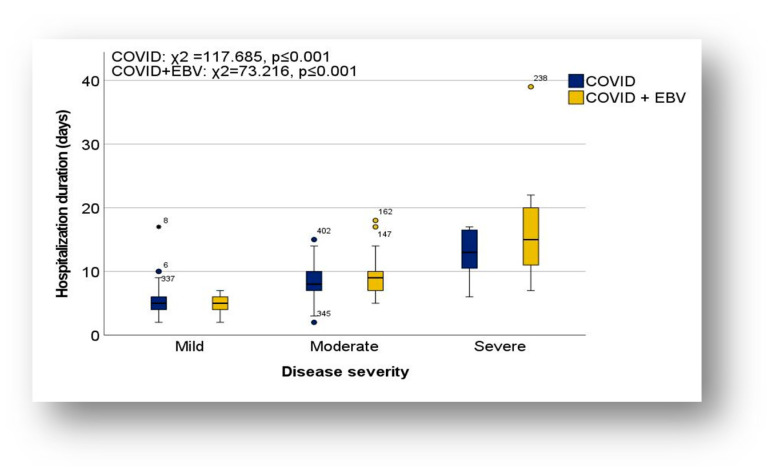
Hospitalization duration (days) by disease severity of illness in the COVID-19 and COVID-19 + EBV subgroups. The figure illustrates how disease severity correlates with length of stay in each subgroup.

The association between severity of illness and duration of hospitalization was assessed using the Kruskal–Wallis H test and the Dunn test with Bonferroni correction. The results showed a significant increase in the duration of hospitalization between mild and moderate or severe cases in both COVID-19 and COVID-19 + EBV subgroups (Figure 4). No significant differences were observed between moderate and severe cases; however, in general, severe cases had a longer duration of hospitalization than moderate cases (COVID-19 subgroup: H = 117.685, *p* ≤ 0.001; COVID-19 + EBV subgroup: H = 73.216, *p* ≤ 0.001).

In mild forms, the duration of hospitalization ranged from 2 to 17 days in the COVID-19 subgroup, with a mean of 5.02 days (standard deviation 1.96; CI; [4.68;5.37]) and a median of 5 days. In the COVID-19 + EBV subgroup, the maximum duration was 7 days, with a mean duration of 5.02 days (standard deviation 1.33; CI; [4.64;5.40]) and a median of 5 days.

In the moderate cases, the duration of hospitalization was between 2 and 15 days in the COVID-19 subgroup (mean, 8.09 days; standard deviation, 2.34; CI; [7.72;8.47]; median, 8 days), and between 5 and 18 days in the COVID-19 + EBV subgroup (mean, 8.89 days; standard deviation, 8.89; CI; [8.21;9.56]; median, 9 days). In severe forms, the duration of hospitalization was longer (minimum 6 days), with a mean duration of 12.86 days, (standard deviation, 4.14; CI, [9.03; 16.69]) and a median of 13 days in the COVID-19 subgroup, and 17.22 days (standard deviation, 17.22; CI, [9.90; 24.54]) and a median of 15 days in the COVID-19 + EBV subgroup.

##### Duration of Hospitalization and Relationship with Biomarkers

The relationship between the duration of hospitalization and the level of biomarkers (CRP, ESR, D-dimer, and potassium) was assessed using the Spearman correlation coefficient. Application of this coefficient identified direct correlations of weak intensity between duration of hospitalization and CRP (rs = 0.244, *p* ≤ 0.001), ESR (rs = 0.230, *p* = 0.006), serum potassium (K) (rs = 0.222, *p* = 0.001), and a direct correlation of moderate intensity between duration of hospitalization and D-dimer value (rs = 0. 426, *p* = 0.001). These results suggested that, for patients in the COVID-19 subgroup, the duration of hospitalization increased as the CRP, ESR, D-dimer, and potassium levels were higher. These observations were supported by applying Mann–Whitney U and Kruskal–Wallis H tests followed by post hoc analysis. Thus, the mean duration of hospitalization was significantly longer in children with increased CRP compared to those with normal CRP (7.45 vs. 6.23 days, Mann–Whitney U test: U = 11412.500, Z = 3.420, *p* ≤ 0.001). An increase in the duration of hospitalization was also observed in children with increased D-dimer compared to those with normal values (mean duration 8. 68 days vs. 6.04, Mann–Whitney U test: U = 634.500, Z = 2.833, *p* = 0.005). Similar situations were noted in patients with increased serum potassium values, compared to those with normal values, where a significant increase in the duration of hospitalization was observed (7.74 days vs. 6.63 days, Kruskal–Wallis H test followed by post hoc analysis: H (2) =8.509, *p* = 0.014).

**Figure 5:**
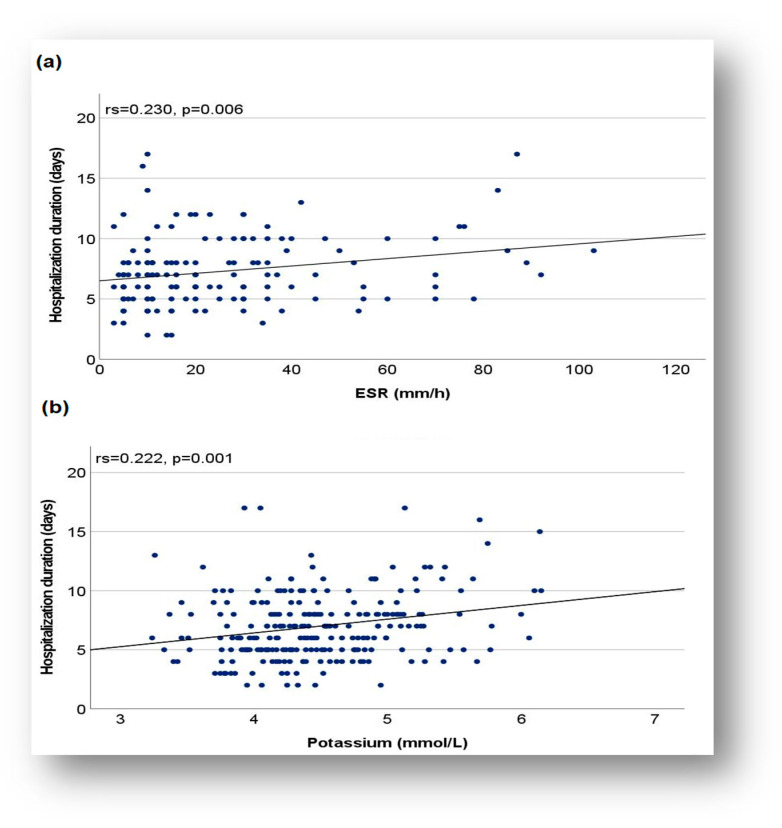
(**a**,**b**) Distribution of cases according to hospitalization duration (days) and biological markers in the COVID-19 subgroup. The figure illustrates a positive correlation, along with longer hospital stays observed at higher ESR and potassium levels.

Regarding the graphical representation, although it showed a monotonous correlation between the duration of hospitalization and biological parameters such as ESR and serum potassium, the regression line with an ascending orientation indicated a trend of increasing the duration of hospitalization as patients had higher values, above normal limits, for these markers (Figure 5a,b).

An aspect noted in the COVID-19 subgroup was the absence of a statistically significant association between the duration of hospitalization and vitamin D (*p* = 0.945). Thus, the mean length of hospitalization was 7.10 days for patients with low vitamin D levels, 6.63 days for patients with optimal vitamin D levels, and 8.69 days for those with high levels of 25-OH vitamin D, showing no significant differences (Kruskal–Wallis test H, H (2) = 3.239, *p* = 0.198). On the other hand, the statistical analysis related to the three categories of length of hospitalization (acute, 1–7 days; subacute, 8–14 days; and chronic, >14 days) revealed that only patients with high vitamin D levels had a hospitalization duration of more than 14 days. In these cases, the Chi-square test indicated significant differences between patient groups, depending on vitamin D level (low/optimal/high) and length of hospital stay (χ^2^ = 19.519, *p* ≤ 0.001). These differences were not noticed in the COVID-19 + EBV subgroups.

The analysis of the subgroups also showed a significant increase in the length of hospitalization in children with increased platelet values (thrombocytosis), compared to those with normal platelet values. Specifically, the average duration of hospitalization was 7.67 days for children with thrombocytosis in the COVID-19 subgroup, compared to 6.46 days for those with normal platelet values. In the COVID-19 + EBV subgroup, the mean duration was 10.42 days for children with increased platelet values versus 6.58 days for those with normal platelet values (Kruskal–Wallis test followed by post hoc COVID-19 analysis: H = 12.390, *p* = 0.002; COVID-19 + EBV: H = 12.684, *p* = 0.002).

For biological markers such as ferritin (*p* = 0.467), procalcitonin (*p* = 0.242), and serum sodium (*p* = 0.460), no significant associations were observed between these markers and the duration of hospitalization within the COVID-19 subgroup.

In the COVID-19/EBV subgroup, no significant association was observed between the duration of hospitalization and biological markers, including CRP (*p* = 0.06), ESR (*p* = 0.059), ferritin (*p* = 0.633), procalcitonin (*p* = 0.947), D-dimers (*p* = 0.212), Na (*p* = 0.220), and vitamin D (*p* = 0.796).

### 3.3. Variations in Metabolic and Biochemical Status in the Clinical Context

#### 3.3.1. Distribution of Vitamin D in COVID-19 and COVID-19 + EBV Subgroups

**Figure 6:**
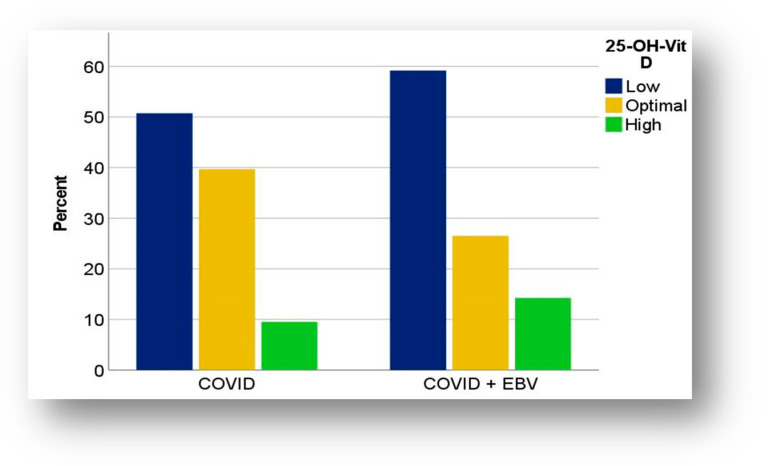
Distribution of 25-OH vitamin D levels across the COVID-19 and COVID-19 + EBV subgroups. While visual differences are noted between groups, no statistically significant association was found (*p* = 0.239).

Analysis of vitamin D (25-OH vitamin D) levels showed notable trends in its distribution, but did not identify a statistically significant association between vitamin D levels and the patient’s immune status. In both subgroups, vitamin D values were often below the optimal limit, with hypovitaminosis D being more common in children with COVID-19 + EBV coinfection (59.18%) compared to those with post-COVID-19 infection status (50.74%) (Figure 6).

Moreover, patients with coinfection experienced severe vitamin D deficiency more frequently (16.33%) than those in the COVID-19 subgroup (7.35%). The minimum value recorded in the COVID-19 + EBV cohort was 8.77 ng/dL, with a median value of 28.30 ng/dL, while in the COVID-19 cohort, it was 12 ng/dL, with a median value of 29.30 ng/dL (Table 3). Approximately 1 in 10 children, in both subgroups, had increased vitamin D levels, with maximum values of 113 ng/dL in the COVID-19 subgroup and 80.10 ng/dL in the coinfection subgroup.

Regarding the age subcategories, a slight difference was observed between the variations in vitamin D (25-OH vitamin D) levels. In the COVID-19 subgroup, the median age value in patients with hypovitaminosis D was 7 years (IQR: 3–13 years) compared to 9 years (IQR: 5.5–13.5 years) in the subgroup with coinfection.

At the same time, a slight tendency for female patients to present with lower levels of 25-OH vitamin D more frequently compared to boys (60.94% versus 41.67%) was observed in the COVID-19 subgroup. In the male patients, normal values of vitamin D were more frequently noted (50%) compared to girls (28.13%). This association was assessed as statistically significant in the COVID-19 subgroup (Chi-square test, χ^2^ = 6.804; φ = 0.224; *p* = 0.033).

On the other hand, in the subgroup with coinfection, it was noted that boys had lower levels of vitamin D more frequently (69.23%), compared to females (52.17%), and optimal values were observed more often in female patients (17.40%), compared to males (15.38%). In the subgroup with coinfection, there was no association between vitamin D levels and gender.

Another noteworthy aspect was that hypervitaminosis D was more commonly found in younger patients, with a median age of 2.5 years (IQR: 1.25–5.25 years) in the COVID-19 cohort, compared to 1 year (IQR: 0.25–6.5 years), in the cohort with coinfection. Gender-related hypervitaminosis was more frequently observed in girls, both in the COVID-19 subgroup (30.43%) and in the subgroup with coinfection (10.94%).

Concerning the temporal distribution of cases with vitamin D variations outside the reference range, several distinct aspects were noted in the two subgroups. Specifically, hypovitaminosis D was detected in the COVID-19 subgroup, predominantly during the cooler seasons (69.12%), characterized by lower temperatures and shorter periods of sunshine (September–February). In contrast, in the COVID-19/EBV subgroup, only 41.38% of cases with hypovitaminosis D were observed in September–February, with the remaining cases were observed in the months with higher temperatures and more extended periods of sunshine (March–August).

A notable aspect in the case of hypervitaminosis D was its dominant presence in the cooler seasons, from September to February, and very rarely in the months with higher temperatures and more extended periods of sunshine (March–August). Thus, hypervitaminosis D was observed in only 21.43% of cases with post-COVID-19 infection status and in 28.57% of patients with coinfection during periods with abundant sun and higher temperatures. We mentioned that Romania is positioned at 45°latitude north, and the climate is temperate, with four seasons, meaning that excessive exposure to the sun can only be achieved from June to August.

##### Vitamin D and Biological Parameters in COVID-19 and COVID-19 + EBV Subgroups

**Figure 7:**
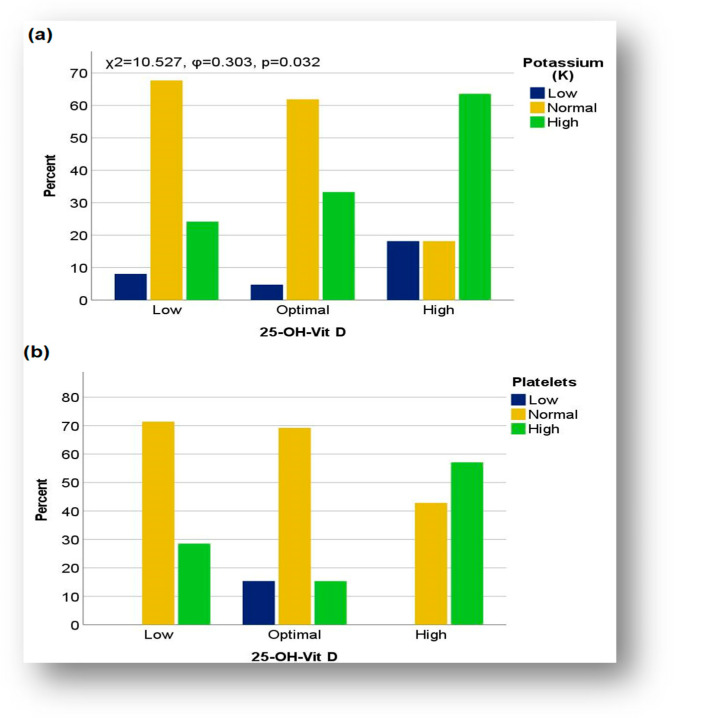
(**a**,**b**) Distribution of cases by 25-OH vitamin D status and biological parameters: potassium levels in the COVID-19 subgroup (**a**) and platelet count in the COVID-19 + EBV subgroup (**b**). No statistically significant associations were identified (*p* = 0.066).

The application of the Chi-square test revealed a statistically significant association between the level of 25-OH vitamin D and serum potassium disorders (χ^2^ = 10.527, φ = 0.303, *p* = 0.032), specifically in the subgroup with post-COVID-19 infection status. Children with high levels of vitamin D had a moderate tendency to have abnormal potassium levels more frequently, compared to those with optimal or low levels of vitamin D. In children with hypervitaminosis D, the percentages of cases with normal or low potassium values were similar. In contrast, children with optimal or low levels of vitamin D had normal potassium levels more frequently (Figure 7a).

Although no statistically significant association was observed in the coinfection group, increased potassium levels were more common in the context of hypervitaminosis D (50%) compared to children with optimal levels (27.27%) or those with hypovitaminosis (25%). Notably, low potassium values (25%) were found exclusively in children with hypovitaminosis D, whereas children with optimal vitamin D levels had predominantly normal potassium levels (72.72%).

Another remarkable aspect in the COVID-19 + EBV subgroup was those children with hypervitaminosis D presented considerably more frequently with increased levels of platelets (57.14%) compared to those with hypovitaminosis D (28.57%), or optimal levels of 25-OH vitamin D (15.38%). In contrast, low platelet counts were found exclusively in children with optimal vitamin D levels (Figure 7b).

##### Vitamin D in the Context of the Severity of the Illness

In the COVID-19 subgroup, low vitamin D levels were more commonly associated with more severe forms of the disease. Specifically, 66.67% of children with hypovitaminosis D had moderate forms of the disease, while 33.33% had mild forms. In contrast, among children with optimal or high levels of vitamin D, the distribution of mild forms of the disease was balanced (44.44% versus 46.15%). Moderate forms were more common in patients with optimal vitamin D levels (55.56%) compared to those with high levels of 25-OH vitamin D (46.15%). Severe forms of the disease were observed only in patients with hypervitaminosis D; their presence was noted in 7.69% of cases, with the predominance of gastrointestinal disorders followed by respiratory and neurological ones. The application of a Chi-square test indicated a statistically significant, low-intensity association between vitamin D levels and the severity of clinical manifestations (χ^2^ = 11.708, φ = 0.293, *p* = 0.020). This association was observed only in the subgroup with post-COVID-19 infection status.

In relation to clinical manifestations or the presence of inflammatory syndrome, vitamin D levels were not statistically significantly associated with symptoms such as respiratory, gastrointestinal, or cardiac, nor with inflammatory markers (CRP, ESR, ferritin, and procalcitonin), in the studied subgroups.

#### 3.3.2. Serum Electrolyte Variations in COVID-19 and COVID-19 + EBV Subgroups

The analysis of the subgroups did not reveal statistically significant differences in the level of electrolytes such as sodium or serum potassium.

In the COVID-19 subgroup, most patients (84.58%) had normal serum sodium (Na) levels, with variations between 130 and 167 mmol/L. A slight increase in sodium levels was noted in 9.58% of cases, while a decrease in serum level was observed in only 5.83% of patients. Serum sodium variations were detected predominantly in female patients; hyponatremia was observed in 71.43% of cases and hypernatremia in 62.07% of cases. Regarding the age of the patients, hyponatremia was noted in the 0–9 years age range, with a median value of 4 years (IQR: 1–6 years), while hypernatremia was detected in the 1–17 years age range, with a median age value of 7 years (IQR: 4–11 years).

Regarding the distribution of serum sodium in the COVID-19 + EBV subgroup, 82.98% of patients had normal values. Serum sodium levels ranged from 129 mmol/L to 148 mmol/L in this subgroup (Table 3). Of the total number of patients with coinfection, 11.70% had low levels of serum sodium, while in 5.32% of cases, increased levels were observed. Hyponatremia was frequently noted in male patients (61.11%). Low serum sodium levels were detected in patients aged 0–16 years, with a median age of 8.5 years (IQR: 5–10 years). In contrast, hypernatremia was observed in 66.67% of cases in female patients, and the median age value was 4 years (IQR: 0.25–8.5 years).

Serum potassium analysis revealed a similar distribution between subgroups, with no significant differences (*p* = 0.905). In both subgroups, most patients had normal potassium values (54.81% in the COVID-19 subgroup and 52.17% in the COVID-19 + EBV subgroup). Among the pathological variations in serum potassium, hyperkalemia was most frequently observed in 33.47% of cases within the post-COVID-19 infection status subgroup and in 35.87% of cases with coinfection. An aspect worth noting was that hyperkalemia was the most common electrolyte disorder, present in both subgroups, while low potassium values were the least observed, both in the COVID-19 (11.72%) and in the COVID-19 + EBV subgroups (11.96%).

Increased potassium levels were more commonly observed in male patients (58.46%, *n* = 35) in the COVID-19 subgroup, whereas in the coinfection subgroup, they were noted in female patients (55.26%, *n* = 21).

Hyperkalemia was noted in both subgroups, in the age range 0–17 years, with a slight difference in the median age value. The median value was 2 years (IQR: 1–4 years) in the COVID-19 subgroup and 1.5 years (IQR: 1–6 years) in the COVID-19 + EBV subgroup.

##### Hydro-Electrolyte Disorders and Variations in Coagulation Markers

In our study, we also aimed to explore potential associations between altered electrolyte status and thrombotic risk in the context of post-COVID-19 infection and SARS-CoV-2 coinfection in the pediatric population. Since viral infections can generate a systemic inflammatory response, with direct implications for the coagulation cascade, D-dimer has been selected as a marker of fibrinolytic activity and a potential early indicator of a prothrombotic status.

The analysis did not reveal significant associations between D-dimers and sodium (*p* = 0.447) or potassium (*p* = 0.820) levels in the COVID-19 + EBV subgroup. In contrast, among children with post-COVID-19 infection status, a statistically significant association of moderate intensity was observed between the increase in D-dimers and abnormal sodium levels (Chi-square test, χ^2^ = 7.074; φ = 0.352; *p* = 0.029). In all cases with low and elevated sodium values, D-dimer levels were increased. In contrast, in patients with normal serum sodium values, only 55.32% had elevated D-dimer values. In addition, all cases with normal D-dimers had normal sodium values. As for potassium, in the COVID-19 group, there were no significant associations between the potassium level and the post-COVID-19 infection status (*p* = 0.355).

## 4. Discussion

The present research contributes to our understanding of the complexity of SARS-CoV-2 and Epstein–Barr infections by investigating key aspects of the clinical and biological course of pediatric patients, with a particular focus on variations in serum electrolytes and vitamin D (25-OH vitamin D) levels. Data analysis revealed several patterns that provided valuable insights into the significant interdependencies between these factors and parameters such as biological markers, duration of hospitalization, and severity of illness.

Infection with SARS-CoV-2 is associated with a broad spectrum of clinical manifestations, including not only respiratory system damage, but also other organs such as the gastrointestinal tract and kidneys [34,35]. Scientific research has suggested that the interaction of the virus with the gut microbiota plays a vital role in the development of gastrointestinal disorders and electrolyte imbalances [36]. In addition, due to the significant presence of ACE-2 receptors on podocytes and tubular epithelial cells, SARS-CoV-2 can induce renal dysfunction, negatively influencing water and electrolyte balance [37]. These complications can exacerbate acute respiratory distress syndrome (ARDS), contributing to an increased risk of cardiac damage [38], prolonged hospitalization in severe cases, and, in some cases, increased risk of death. Moreover, recent research highlights a distinct interrelationship between vitamin D concentration and COVID-19 infection. Vitamin D deficiency may be a trigger for ARDS, while optimal levels may exert immunomodulatory and antimicrobial effects by inhibiting cytokines [39,40]. Calcitriol, a vitamin D agonist, has also been shown to protect against acute lung injury by upregulating the expression of components of the renin–angiotensin system, including ACE 2, in lung tissue [20].

Regarding the possibility of a relationship between SARS-CoV-2 and Epstein–Barr virus, a multi-omics longitudinal study indicated that EBV viremia level might be a significant risk factor in the development of prolonged COVID-19 [41]. In a study investigating the interaction between EBV and COVID-19, conducted in Wuhan, China, it was found that 55.2% of patients hospitalized with COVID-19 had IgM antibodies against EBV (VCA), indicating possible reactivation of Epstein–Barr virus [14]. In addition, a correlation between EBV DNA load and COVID-19 severity has been reported. More than 50% of patients with severe COVID-19 hospitalized in the intensive care unit (ICU) showed EBV reactivation or a significant increase in antibodies against EBV, with Epstein–Barr viremia detectable in the plasma of critically ill SARS-CoV-2 patients [42,43]. Thus, it has been hypothesized that SARS-CoV-2 infection tends to reactivate EBV at a higher rate than in non-COVID-19 patients, and that the link between the two viruses significantly contributes to a deterioration in general condition or significantly increased mortality compared to EBV-negative patients [44,45].

### 4.1. Vitamin D Levels and Distribution in COVID-19 and COVID-19 + EBV Subgroups

In this study, we evaluated the 25-OH vitamin D distribution in two subgroups of children: one with post-COVID-19 infection status and another with SARS-CoV-2 and EBV coinfection. Although there is not sufficiently robust evidence to establish a direct relationship between vitamin D and post-COVID-19 infection effects in children, there are several studies suggesting that vitamin D may modulate the immune response, with a protective role against respiratory infections [46]. It has also been shown that pediatric patients with low vitamin D levels are more susceptible to respiratory infections. Thus, it is hypothesized that an optimal vitamin D level may modulate the inflammatory response by reducing the activity of interleukin-6 (IL-6) and interferon gamma (IFNγ), two predictors of unfavorable clinical outcome in severe COVID-19 [47]. Additionally, vitamin D deficiency has been correlated with the overexpression of Th1-type cytokines, suggesting ineffective regulation of inflammation [48].

Clinical trials, including those in adults, have demonstrated that vitamin D deficiency contributes to a weaker immune response, leading to unbalanced inflammation and exacerbating disease progression in cases of COVID-19 [21].

#### 4.1.1. Hypovitaminosis D

In our study, more than 50% of patients had vitamin D levels below the optimal limit. This observation is consistent with data in the literature that indicates a similar prevalence of vitamin D deficiency among patients with COVID-19. For example, in an extensive study of 8176 patients, it was found that about 65% of patients with COVID-19 suffered from vitamin D deficiency [49].

Most importantly, our study focused on assessing vitamin D levels in the post-infection period, when the immune response is in a phase of recovery and regulation. Still, some immune and inflammatory changes may persist. During this period, even if the acute infection has been overcome, specific immunologic susceptibility can influence vitamin D levels and how the body responds to possible secondary infections or residual inflammation.

Our results indicated a more frequent vitamin D deficiency in patients with COVID-19 + EBV coinfection (59.18%) compared to those with post-COVID-19 infection status (50.74%). Moreover, severe vitamin D deficiency (less than 10 ng/dL) was more common in the coinfected subgroup (16.33%) compared to the post-infection subgroup (7.35%). The differences in the prevalence of hypovitaminosis D between the two subgroups could be explained by post-infection immunologic status and extrinsic or intrinsic factors predictors of vitamin D deficiency [50].

Vitamin D deficiency was observed in all age groups, in both subgroups. Although there was a slight difference between the two subgroups, most patients with hypovitaminosis D fell into the age subgroup of 6–11 years. This result emphasizes the fact that most of the patients had similar physiological and pathological characteristics in the same age range.

In the literature, age has been identified as an important factor in the prevalence of vitamin D deficiency, with a higher risk observed at younger ages [50]. This finding is also supported by another study, which reported that vitamin D insufficiency or deficiency is present in all age groups [51]. In this regard, our observations are also congruent with the existing literature, confirming that hypovitaminosis significantly affects children of all age groups, especially in the post-infectious period, where immunologic function may be impaired.

Another relevant aspect is that most cases of coinfection occur in the cold season, characterized by low temperatures and limited sun exposure, which is an additional risk factor for vitamin D deficiency [14].

Although hypovitaminosis D is frequently encountered at younger ages, as reported in the literature [52], in the case of COVID-19 + EBV coinfection, the association with the cold season may be an important contributing factor in regard to the higher prevalence of vitamin D deficiency. Thus, even though vitamin D deficiency is traditionally associated with younger ages, the context of the cold season and the possible exacerbation of vitamin D deficiency during this period could provide an additional explanation for the relatively higher prevalence of deficiency among patients with coinfection.

Regarding the gender distribution, we noted a higher prevalence of hypovitaminosis D among female patients (60.94%) in the post-COVID-19 infection-status subgroup. A statistical analysis of the two variables indicated a statistically significant association (*p* = 0.033). These results are consistent with the literature, which reported that being female may be one of the risk factors for vitamin D deficiency [50]. However, in the cohort with COVID-19 + EBV coinfection, hypovitaminosis was more frequently noted in males (69.24%) compared to females (52.17%), indicating that, in the context of coinfection, other immunologic or physiologic factors may influence vitamin D levels, such as hormonal differences and immune response between sexes. Although there is no definitive explanation, the literature suggests that male patients may be more vulnerable to coinfections, particularly in the context of viral infections, due to immune differences between the sexes [53,54].

Although hypovitaminosis D has been noted quite frequently, statistical analysis did not reveal a significant association between low vitamin D levels and post-COVID-19 infection status. Similarly, despite the ability of EBV to modulate the host immune response [55,56], no statistically significant association was observed between low vitamin D levels and SARS-CoV-2/EBV coinfection in our study. In most cases in the coinfected group, a post-EBV infection status (79.34%) without a significant active immune response was observed. Only in 4.13% (*n* = 5) of cases, hypovitaminosis D and recent active infection (positive anti-EBV IgM) were detected simultaneously. This might suggest that in most cases, the Epstein–Barr virus was not an active pathogen, but rather a latent virus, influencing the interactions with vitamin D metabolism and the immune response.

#### 4.1.2. Hypervitaminosis D

Although hypervitaminosis D is regarded as a rare condition in the general population, the analysis carried in this study revealed a surprisingly high prevalence among young children with post-COVID-19 infection or SARS-CoV-2/EBV coinfection. This finding, unusual in a pediatric setting, justifies exploring the possible mechanisms involved in the occurrence of hypervitaminosis D in these cases. Furthermore, the inclusion of this topic in the discussion is motivated by the identification of contextual and behavioral factors, such as uncontrolled vitamin D supplementation during the pandemic, but also by the possible involvement of chronic inflammatory processes or metabolic dysfunctions induced by viral infections in the disruption of vitamin D metabolism. Based on these observations, we analyzed in detail the characteristics of the identified cases with hypervitaminosis D in our cohort to better understand the mechanisms and factors that contribute to this situation.

Hypervitaminosis, defined as a value greater than 50 ng/dL, was observed in our study more frequently among young children, especially in the subgroup with SARS-CoV-2/EBV coinfection. The median age in the COVID-19 cohort was 2.5 years, compared with 1 year in the coinfected cohort. Although it has been stated that a younger age is a predisposing factor for vitamin D deficiency [45], our study found that the age of patients was lower in cases with hypervitaminosis D than in cases with low vitamin D levels. Considering that in most cases the increased vitamin D level did not occur because of prolonged exposure to the sun, with more than 70% of the cases occurring during colder periods of the year, we can consider that certain factors contributed to an increase in vitamin D level above the optimal value.

A first factor could be excessive vitamin D supplementation, which is predominantly seen during periods with short sunshine exposure. Epidemiologic studies have shown that in temperate regions such as Romania, vitamin D supplementation in the cold season is essential [57]. Moreover, recent research has shown that, in the context of the COVID-19 pandemic, many of the recommendations for vitamin D supplementation were adopted as preventive measures, but without adequate monitoring of plasma levels, leading to hypervitaminosis [58]. This observation could be due to prophylactic behaviors such as excessive vitamin D supplementation, especially during colder periods, when sun exposure is limited. The literature also suggests that, ideally, vitamin D supplementation should be administered according to the patient’s plasma levels and individual needs to avoid the risk of hypervitaminosis [59]. Furthermore, a recent review has revealed that higher doses of vitamin D given without monitoring can cause hypercalcemia and other complications associated with vitamin D toxicity [60].

Prolonged systemic inflammation, induced by SARS-CoV-2 and EBV, may also contribute to abnormal D metabolism, amplifying the risk of hypervitaminosis [49,61]. Recent studies emphasize that these infections can disrupt the natural balance between vitamin D synthesis and the body’s need to respond to viral attacks, which can lead to significant changes in vitamin D levels. Moreover, viruses such as SARS-CoV-2 and EBV can induce prolonged systemic inflammation [40,62,63], which directly affects vitamin D metabolism. This inflammation leads to an excessive stimulation of 1α-hydroxylase expression in macrophages and other immune cells, resulting in increased vitamin D activity, with additional risks of hypercalcemia [64]. A similar mechanism can be observed in granulomatous diseases, such as tuberculosis, where the conversion of 25-OH vitamin D to 1,25-OH2 vitamin D is exaggerated. In addition, viral infections generated by SARS-CoV-2 or EBV can lead to post-infectious kidney and liver damage [65,66], as well as to dysregulation of the hormonal axis and calcium metabolism [67], increasing the body’s vulnerability to normal vitamin D regulation and increasing the risk of hypervitaminosis D.

An interesting aspect in our study was also the unequal distribution of hypervitaminosis D between the sexes, observed in both subgroups, with a higher prevalence among girls (10.94%) in the COVID-19 subgroup and 30.43% in the coinfected subgroup. Although the literature suggests a higher prevalence of hypervitaminosis D in boys, our study showed a higher prevalence of hypervitaminosis D in girls in both subgroups, suggesting a possible influence of population-specific factors, such as the complex interaction between viral factors and hormonal differences. Thus, simultaneous exposure to two viral infections, such as SARS-CoV-2 and EBV, could amplify this predisposition by indirectly influencing vitamin D metabolism, through changes in the immune response. There is also evidence that male gender may be associated with a stronger immunologic response to certain viral infections, which could explain the incidence of hypervitaminosis D in boys via an enhanced immune response [68]. Furthermore, previous studies have shown a complex, bidirectional interaction between estrogen and vitamin D metabolism. Thus, different levels of estrogen reduce the expression of the CYP24A1 gene involved in vitamin D inactivation and increase the expression of the VDR gene. At the same time, vitamin D has been shown to reduce the expression of aromatase, which converts testosterone to estrogen in immune cells [54].

#### 4.1.3. The Role of Vitamin D in the Context of Disease Severity

The 25-OH vitamin D analysis revealed interesting trends, but did not show a direct and statistically significant association between vitamin D deficiency and the severity of disease manifestations. These results do not fully support observations in the literature that suggest that vitamin D regulates the immune system by stimulating CD8+ T cells, essential for the immune response against viral infections [69]. In a comparative study in children with pneumonia and sepsis, a significant association between low vitamin D levels and more severe forms of respiratory infections was demonstrated (*p* < 0.001) [64]. Other research in adults with COVID-19 also found that vitamin D deficiency was frequently associated with severe disease manifestations, and 74.1% of COVID-19 patients had low vitamin D levels and high severity of infection [70]. The study by Lau et al. also found a correlation between vitamin D deficiency and COVID-19 disease severity in 75% of moderate cases of COVID-19 and in 84.6% of patients in the intensive care unit [71]. These observations suggest that vitamin D may influence disease severity by modulating the immune response.

In contrast to previous studies, our analysis did not reveal a significant correlation between low vitamin D levels and disease severity in children. More specifically, although a trend of association between hypovitaminosis D and moderate forms of disease was found descriptively in the subgroup with post-COVID-19 infection status (66.67%), this was not observed in the subset with COVID-19 + EBV coinfection.

However, our analysis revealed a significant association between elevated 25-OH vitamin D levels (hypervitaminosis) and severe forms of disease (Chi-square, χ^2^ = 11.708; φ = 0.293; *p* = 0.020), but only in the subgroup with post-COVID-19 infection status. This indicates that excess vitamin D might influence the clinical manifestations differently, possibly related to the adverse effects of excess vitamin D, such as hypercalcemia and electrolyte disturbances [72,73,74]. Thus, in addition to vitamin D deficiency, hypervitaminosis D may be another important factor influencing disease severity in acute or previous SARS-CoV-2 infection.

These observations suggest that vitamin D plays a vital role in supporting the immune response, with an impact on the severity of COVID-19 disease in the post-infectious state. However, other associated factors may contribute to disease progression. In addition, the fact that we did not identify a clear link between vitamin D deficiency and the severity of respiratory manifestations suggests that further studies addressing the complexity of these interactions and including a wide range of clinical and biological variables are needed.

#### 4.1.4. Vitamin D Levels and Biomarkers Variations

It is essential to highlight that the relationship between vitamin D levels and relevant biological parameters, such as potassium and platelet counts, may offer further insights into the pathophysiological interactions associated with SARS-CoV-2 infections or coinfections.

In this regard, a trend of increasing potassium abnormalities was noted in patients with hypervitaminosis D in both subgroups, suggesting a possible link between electrolyte imbalances and vitamin D levels (*p* = 0.032). Moreover, in the subset with post-COVID-19 infection status, the frequent presence of common manifestations, such as gastrointestinal disorders (abdominal pain, nausea, and vomiting), neurological symptoms (paresthesia and muscle weakness), and, to a lesser extent, cardiovascular issues, supports the interrelation between electrolyte imbalances such as hyperkalemia and elevated vitamin D levels. It is well known that the heart undergoes a series of structural and functional adaptations after intense and repetitive exercises [75]. The concomitant presence of these manifestations emphasizes the significant impact of these disorders on the clinical course of patients and their link with more severe forms of the disease. These observations are consistent with results reported in the literature on the impact of vitamin D on mineral metabolism, bone health, and immune regulation [76,77].

Regarding the COVID-19 + EBV subgroup, our study revealed a variable distribution between vitamin D and serum potassium levels, providing a complex picture of the interaction between these two markers.

In cases with hypovitaminosis D, the distribution of serum potassium levels seems more dispersed, with changes in both directions (hyperkalemia, −25%; and hypokalemia, −25%), compared with cases with hypervitaminosis D, where the deviation was only upward (50% hypokalemia). This may suggest a possible link between vitamin D deficiency and decreased potassium levels, although the causal relationship cannot be established from these observations alone.

Relating the result of this study to the scientific literature data, it seems to be supported by studies that have shown that hypovitaminosis D may affect electrolyte metabolism, including potassium, to a greater extent than hypervitaminosis D, due to the role of vitamin D in the regulation of renal function and electrolyte homeostasis [76]. As for the increased vitamin D levels and the impact on potassium metabolism, the link between these two markers is less clear and depends on risk factors such as the severity of hypervitaminosis and the presence of comorbidities [78].

Another aspect noted in our study was the incidence of thrombocytosis in the context of hypervitaminosis D. Although increased platelet counts are not typical in EBV or COVID-19 infection, thrombocytosis was more frequently noted in our study compared to thrombocytopenia. The complex interactions between the viruses involved could explain its presence in the COVID-19 + EBV subgroup and the common effects of EBV and SARS-CoV-2 on the immune system.

Typically, thrombocytosis in children is reactive, often occurring during the recovery period from infection or inflammation. A wide range of cytokines, such as IL-3, IL-11, and granulocyte–macrophage colony-stimulating factors, as well as thrombopoietin and IL-6, are involved in stimulating platelet production in response to infection [79]. In addition, the Epstein–Barr virus is known for its ability to induce an exacerbated response, which can lead to activation of the hemostasis system and thus thrombocytosis. In this context, the presence of COVID-19 + EBV coinfection could modulate vitamin D metabolism and inflammatory responses differently, leading to exacerbation of biological and clinical manifestations, including thrombocytosis, compared with singular viral infection. The combination of the two infections could act as an additive or synergistic factor in increasing the risk of thrombocytosis, significantly influencing the course of the disease.

These trends not only emphasize the diversity of clinical manifestations, but also highlight the interdependence of these biological factors, which may influence the severity of disease progression in coinfected patients.

### 4.2. Serum Electrolyte Variations in the Analyzed Subgroups

Our study identified significant differences in the distribution of serum sodium and potassium levels between the patients with post-COVID-19 infection status and COVID-19 + EBV coinfection.

Hydro-electrolyte disturbances are a common complication in viral infections, having a significant impact on serum hydro-electrolyte balance and osmolarity. These imbalances can influence kidney function and the body’s electrolyte-regulation mechanisms [24]. Hyponatremia is, in particular, one of the most common electrolyte abnormalities found in viral infections, being particularly reported in SARS-CoV-2 infections [80], and its critical values have often been associated with increased mortality or morbidity in severe forms of the disease [81]. Also, in the pediatric population, hyponatremia has been described as the most common electrolyte dysfunction observed in children under 4 years of age, often correlated with gastrointestinal, renal, and endocrine manifestations [82].

In our study, we noted significant differences in the distribution of serum sodium and potassium levels between the patients with post-COVID-19 infection status and SARS-CoV-2/EBV coinfection. Such variations may reflect a different pathophysiological response, depending on the type and severity of the viral infection, as well as the host immune response. These observations align with the existing literature, which emphasizes the complex impact of viral infections on hydro-electrolyte metabolism [83].

A notable aspect was the distribution of hyponatremia in patients with much older ages than those described in the literature, with the median age value being 8.5 years (IQR: 5–10 years) in the coinfected subgroup. These aspects might suggest that SARS-CoV-2/Epstein–Barr coinfection might influence the hydro-electrolyte balance differently in older children, possibly due to a more complex immunologic impact or to the interaction between the two infections, which might amplify the electrolyte imbalances already present in the context of SARS-CoV-2 infection. These observations are consistent with previous studies emphasizing that this association may be related to pathophysiological mechanisms such as syndrome of inappropriate antidiuretic hormone secretion (SIADH), which may be exacerbated by severe inflammation induced by viral coinfection [83].

Moreover, a higher frequency of hyponatremia was observed in the COVID-19 + EBV subgroup (11.70%) compared to the COVID-19 subgroup (5.83%), suggesting a possible synergistic effect between SARS-CoV-2 and Epstein–Barr virus, which could amplify electrolyte imbalances, especially in the context of inflammation induced by viral coinfection.

Given that hyponatremia is most associated with decreased osmolarity, it is advisable to analyze both the serum sodium level and its relationship with urinary and serum osmolarity, as these are critical parameters in determining the mechanism of hyponatremia [84].

Regarding serum potassium, both hyperkalemia and hypokalemia were identified in both cohorts. These variations can be explained by changes in acid–base regulation mechanisms and renal function, which are frequently altered in severe viral infections.

Hyperkalemia was noted as the most common hydro-electrolyte disorder, with a similar distribution in both subgroups. The mean age of patients with hyperkalemia was significantly younger compared to patients with hyponatremia: 2 years (IQR: 1–4 years) in the COVID-19 cohort and 1.5 years (IQR: 1–6 years) in the COVID-19 + EBV cohort. The literature suggests that hyperkalemia may occur at younger ages, particularly in cases with leukocytosis, coagulopathies, or incorrect blood sampling procedures. On the other hand, hyperkalemia occurs in the context of kidney disease at ages older than 4 years [85].

Another aspect worth noting was the distribution of hyperkalemia, which was more frequent in male patients in the COVID-19 subgroup. This result is like studies in which being of the male sex has been reported to be a risk factor for hyperkalemia [86]. In contrast, the presence of hyperkalemia, more frequent in females, in the coinfected subgroup could suggest that the simultaneous action of the two viruses contributed to the alteration of the mechanism regulating serum potassium levels. A first aspect could be the respiratory manifestations and the more frequent presence of polyuria in this subgroup. However, a correlation between hyperkalemia and these clinical manifestations has not been identified, although studies state that patients with hyperkalemia may manifest weakness, paralysis, and even respiratory failure [87].

Elevated potassium levels also correlated with complications noted in coinfected cases, such as renal damage or thrombocytosis associated with hypervitaminosis D. The results complement a recent study, which showed that hyperkalemia with values exceeding the critical threshold in the context of acute or recent SARS-CoV-2 infection was frequently associated with additional complications [88].

Given that electrolyte disturbances, such as hyponatremia or hypernatremia, can indirectly influence inflammatory and hemostatic processes, we found it appropriate to evaluate the relationship between these imbalances and D-dimer levels. Our objective was to identify possible patterns of association that could enhance the understanding of subclinical vascular risks in children with acute or post-viral diseases.

Moreover, analyzing this relationship offers an exploratory framework for stratifying thrombotic risk based on routine biochemical parameters, which are easily accessible in pediatric clinical practice. Thus, the rationale for including D-dimer in this context stems from the hypothesis of a potential common pathogenic mechanism between electrolyte imbalance and hypercoagulability observed in some pediatric cases with SARS-CoV-2, with or without EBV coinfection.

The research carried out emphasizes the complexity of pathophysiological mechanisms involved in the electrolyte and coagulation disturbances found in multiple viral infections. Although significant correlations were observed between hyponatremia and increased D-dimer in patients with post-COVID-19 infection status (χ^2^ = 7.074, φ = 0.352, *p* = 0.029), no significant associations were identified between potassium and coagulation markers in any of the subgroups. These data suggest that in SARS-CoV-2 infection, electrolyte disturbances may influence coagulation activity, increasing the thrombotic risk. Previous studies have already emphasized the importance of monitoring and management of coagulopathy in COVID-19 patients to prevent complications that may arise in the context of abnormalities such as dyselectrolytemias [89].

In pediatrics, such associations are still poorly understood, and identifying significant correlations between hyponatremia or hypernatremia and elevated D-dimer levels may provide valuable insights for early clinical management and monitoring of vascular complications.

### 4.3. Inflammatory Markers in the Context of Immunologic Status

The presence of COVID-19 + EBV coinfection significantly altered the inflammatory profile of the patients, showing a more intense activation of the inflammatory response and coagulation compared to SARS-CoV-2 infection alone.

Inflammatory markers analysis revealed a significant correlation between CRP and coinfection, as well as a substantial presence of the acute-phase reactants ESR and ferritin, with values significantly elevated above the reference range. This suggests that coinfection may result in a more pronounced inflammatory response than single infection. Also, C-reactive protein, a primary marker of acute inflammation, with significantly elevated levels, is associated with systemic activation of the immune response in cases of viral infections or coinfections [90]. In clinical trials, CRP is commonly used to monitor the inflammatory process and the severity of infections. In coinfections, this marker may reflect an enhanced immune response, given the synergistic effects of the two viruses on the immune system.

In addition, ESR is a non-specific indicator of inflammation, and its elevated levels are commonly found in chronic infectious diseases such as EBV infections [91], an aspect also noted in our study, predominantly in the coinfected subgroup. Moreover, the inflammatory marker profile could reflect an intense immune response, a consequence of a possible EBV reactivation.

Additionally, the concurrent or sequential action of SARS-CoV-2 and EBV infections may trigger the development of autoimmune reactions through the overactivation of the immune system. Thus, in autoimmune diseases, such as systemic lupus erythematosus (SLE), severe systemic inflammation is common, and inflammatory markers such as ESR and CRP can reach significantly elevated values [92,93,94]. In this context, viral infections, particularly those with viruses such as SARS-CoV-2 and EBV, may contribute to the development or exacerbation of clinical manifestations of autoimmune diseases through mechanisms including the formation of immune complexes and the activation of specific inflammatory pathways, such as the cytokine storm.

On the other hand, in the COVID-19 subgroup, although a significant association with inflammatory markers was not observed, a higher frequency of IL-6 and procalcitonin was noted, results that correlated with an acute inflammatory response, related to disease severity and progression to acute respiratory distress syndrome (ARDS) [95,96]. The data reported in the literature refers to COVID-19 viral infections and immunologic reactions marked by pro-inflammatory cytokines. Moreover, recent studies have shown the occurrence of complications after SARS-CoV-2 infection, even in mild or asymptomatic forms of COVID-19 [88,97].

### 4.4. Length of Hospitalization and Relation to Biomarkers

Analysis of the relationship between the duration of hospitalization and biomarkers (CRP, VSH, D-dimer, and potassium) revealed a significant association, thus supporting the hypothesis that systemic inflammation and altered immune responses are determinants of the clinical evolution of SARS-CoV-2-infected patients. Results, such as CRP (rs = 0.244, *p* ≤ 0.001) or D-dimer (rs = 0.426, *p* = 0.001), concerning the correlation between length of hospitalization and biomarkers, are supported by the scientific literature, which indicates that inflammatory markers, such as CRP and ESR, are associated with disease severity and more extended hospitalization in viral infections, including COVID-19 [98]. D-dimers, markers of coagulation activation, have also been identified as markers of thrombosis risk and of a thrombotic evolution of the illness [99].

Similarly, the significant increase in hospitalization length observed in cases with thrombocytosis is consistent with existing data suggesting that platelets play a crucial role in modulating the inflammatory response and complications associated with severe viral infections [100]. The results obtained in patients with abnormal serum potassium levels are also supported by previous research, which has demonstrated the link between electrolyte imbalances and the risk of severe complications in viral infections [97].

In our study, markers such as ferritin, procalcitonin, and hypovitaminosis D showed no significant correlations with length of hospitalization, suggesting that these parameters did not directly influence the clinical evolution of patients with post-COVID-19 infection status. However, an association was noted between hypervitaminosis D and a prolonged length of hospitalization of more than 14 days.

Although scientific literature emphasizes the role of ferritin as a marker for inflammation and immune response, correlations with duration of hospitalization are less consistent, and genetic factors and pre-existing conditions may influence these relationships [101].

Contrary to these results, in the coinfected subgroup, no significant correlations were observed between inflammatory markers and duration of hospitalization. This can be explained by the complex nature of the interaction between the two viruses and the variable responses of the host. While markers such as CRP and ESR are frequently associated with systemic inflammation and more severe outcomes in SARS-CoV-2 infections [101], in the context of coinfection, the immune response may be attenuated or modified due to overlapping pathogenic mechanisms of the two viruses. Specifically, EBV can induce a chronic immune response and alter inflammatory responses in acute infections [102]. Viral coinfections can also lead to relative immunosuppression, thereby reducing the effectiveness of inflammatory responses and altering the way inflammatory markers influence disease progression. In addition, viral interaction in coinfections may modulate how biomarkers correlate with disease severity, sometimes in a more subtle or less predictable manner, which could explain the lack of significant associations in this subgroup compared to the post-COVID-19 infection-status subgroup.

Thus, it is possible that in the subgroup of coinfected patients, other factors will play a more important role in determining the duration of hospitalization than biomarkers.

These observations suggest that in SARS-CoV-2/EBV coinfection, the mechanisms of immune response may be different from those observed in isolated infections, and the biomarkers studied fail to clearly reflect the severity of illness or predict the duration of hospitalization.

Our study has some limitations that should be noted. Firstly, the observational–retrospective nature of the research did not allow for a clear causal relationship to be established, as some of the studied variables may be incomplete or inconsistent. Additionally, the relatively small sample size and geographically limited area may restrict the generalizability of the findings. Although we attempted to include variables relevant to the analysis of immune and electrolyte response, we were unable to control all interfering factors that might influence the results. Another limitation of our study is that the analysis of biomarkers was performed only during hospitalization, without including their assessment after discharge.

This restriction prevented the analysis of their long-term evolution and impact on post-infectious recovery. Moreover, an important aspect of our study is the absence of precise data on the exact timing of SARS-CoV-2 infection in patients. Thus, we could not adequately assess whether patients are at a recent stage of infection or whether they have specific manifestations of post-COVID-19 syndrome, which may significantly impact biomarkers and their evolution. A further analysis of the SARS-CoV-2 variants (Alpha, Delta, and Omicron) would have allowed us to assess how each variant influenced the occurrence of electrolyte and vitamin D imbalances, potentially through specific subcategories.

### 4.5. Future Research

In terms of future research, our study suggests the need for a more detailed analysis of the long-term effects of SARS-CoV-2 infection on biological markers, electrolytes, and vitamin D levels, in the context of COVID-19 post-infectious status. This research could include close monitoring of vitamin D and electrolyte levels throughout the post-COVID-19 recovery period. To better assess the long-term impact on the health of pediatric patients and consider both the risks associated with hypovitaminosis D and hypervitaminosis D, future studies should focus not only on low vitamin D levels, but also on increased vitamin D levels and potential adverse effects on clinical outcomes. The use of artificial intelligence in modern medicine can create predictability patterns in the biological evolution of different markers, electrolytes, and vitamins, thus leading to the optimization of diagnosis and treatment [103].

It is essential to investigate the immunologic and pathophysiological mechanisms involved in SARS-CoV-2/EBV coinfections, given the complexity of the interaction between the two viruses. Extensive longitudinal and clinical studies could contribute to a better understanding of how coinfection influences the long-term course of the disease, as well as the risks associated with EBV reactivation in the context of acute viral infections. It is also necessary to investigate the possible long-term effects of these coinfections on the immune system, electrolyte balance, and vitamin D levels of patients, especially in the context of the possible development of autoimmune diseases or cardiovascular complications.

It is important to further explore the impact of persistent changes in electrolyte and other biological parameters in patients with post-COVID-19 symptoms, particularly to better understand how these changes may contribute to the long-term evolution and management of the illness.

Another further research direction could be the identification of additional biomarkers, allowing for a more accurate monitoring of severity and prognosis in post-COVID-19 syndromes or viral coinfections, given their significant impact on the clinical status of patients. In addition, future studies could explore the impact of virus-specific therapy on the modulation of the immune and inflammatory response, thus providing a better understanding of optimal therapeutic strategies for the treatment of patients with post-infectious status or COVID-19/EBV coinfection.

## 5. Conclusions

The results of this study highlight the importance of an integrated approach in post-infectious pediatric assessment, with a focus on monitoring vitamin D status, electrolyte balance, and inflammatory profile. SARS-CoV-2/EBV coinfection was associated with more pronounced metabolic and immunological disturbances than single SARS-CoV-2 infection, suggesting a complex interaction between viral agents and host response mechanisms.

The observed immunological and metabolic peculiarities, including the prevalence of hypervitaminosis D in the context of a recent viral infection, suggest the need to reassess the current clinical and biological surveillance paradigms.

The findings indicate a significant association between changes in serum potassium and increased D-dimer levels, as well as a statistical relationship between hyperkalemia and hypervitaminosis D, especially in the subgroup with post-COVID-19 status. Although there was no direct correlation between hypovitaminosis D and inflammatory markers, the data supports the hypothesis that vitamin D regulates the immune response and modulates the severity of clinical manifestations.

In an ever-changing post-pandemic landscape, these data can help shape future research directions focused on a deep understanding of the long-term effects of multiple viral infections on pediatric body homeostasis. Specifically, longitudinal and interventional studies could support the development of personalized prevention and treatment strategies tailored to the specific vulnerabilities identified in this population, aiming to mitigate associated risks and improve long-term outcomes.

## Figures and Tables

**Table 1:** Reference values of biological parameters’ levels by age and gender.

Biological Parameters	Age Group	Level Range
Sodium (mmol/L)	<3 years 3–<6 years 6–<16 years >16 years (females) >16 years (males)	135–142 135–142 136–143 137–142 137–143
Potassium (mmol/L)	0–<6 years (females/males) >6 years (females/males)	3.9–4.6 3.8–4.9
25-OH vitamin D (ng/dL)	0–18 years	<20 deficiency 20–30 insufficient 30–50-normal 50–150 increased level >150 toxic level
Anti-SARS-CoV-2 IgG antibodies (AU/mL)	0–18 years	0–10
Anti-SARS-CoV-2 IgM antibodies (AU/mL)	0–18 years	0–10
Anti-EBV(VCA) IgG antibodies (AU/mL)	0–18 years	0–5
Anti-EBV(VCA) IgM antibodies (AU/mL)	0–18 years	0–10
Anti-EBV(EBNA) IgG antibodies (AU/mL)	0–18 years	0–5

**Table 2:** Basic clinical characteristics of the analyzed cases from the two subgroups.

Variables	COVID-19 Subgroup	COVID-19 + EBV Subgroup	*p*-Value
	Categorical variables % (N)		
Disease severity			0.06
Mild	44.91 (128)	41.32 (50)	
Moderate	52.63 (150)	51.24 (62)	
Severe	2.46 (7)	7.44 (9)	
	Numerical variables % (N)		
Hospitalization duration (days)			0.045
1–7 days	62.11 (177)	57.85 (70)	
8–14 days	35.14 (103)	35.54 (43)	
>14 days	1.75 (5)	6.61 (8)	

**Table 3:** Baseline paraclinical characteristics in patients with a post-COVID-19 infection status versus patients with a SARS-CoV-2/EBV coinfection.

Biological Markers	COVID-19 Subgroup		COVID-19 + EBV Subgroup	
	Mean Value (SD; CI: [])	Median	Mean Value (SD; CI: [])	Median
Erythrocyte sedimentation rate (ESR) [2–12 mm/h]	25.44 (21.99; [21.85;29.02])	19	30.92 (24.76; [25.79;36.13])	21.00
C-reactive protein [0–0.5 mg/dL]	2.37 (4.50; [1.83;2.90])	0.63	4.13 (6.64; [2.91;5.33])	1.62
Platelets [150–450 × 10³/µL]	372 (147.57; [354.77;389.24)]	344	378.43 (196.52; [342.75;414.10)]	343.3
Procalcitonin [0–0.5 ng/mL]	0.95 (2.90; [0.23;1.65])	0.13	0.63 (1.57; [0.19;1.05])	0.16
Ferritin [13–68 ng/mL]	98.33 (147.33; [72.35;124.30])	53.51	140.61 (212.30; [90.72;190.50]	54.80
IL-6 [0–17 pg/mL]	48.93 (9.02; [34.56;63.28])	47.39	27.91 (28.50; [0;73.26])	20.45
D-dimers [0–0.55 ng/mL]	1.74 (4.66; [0.54;2.9])	0.62	2.26 (3.91; [1.20;3.32])	0.91
Anti SARS-CoV-2 IgG antibodies [0–10 AU/mL]	70.08 (48.49; [64.42;75.72])	62.22	71.51 (56.55; [61.33;81.69])	64.10
Anti-SARS-CoV-2 IgM antibodies [0–10 AU/mL]	5.36 (38.72; [0.12;10.50])	0.61	3.72 (18.57; [0.02;7.43])	0.46
Anti-EBV(VCA) IgG antibodies [0–5 AU/mL]	--	--	57.24 (39.77; [49.99; 64.49])	46.45
Anti-EBV(VCA) IgM antibodies [0–10 AU/mL]	--	--	13.09 (19.65; [8.51;17.68]	5.00
Anti-EBV(EBNA)IgG antibodies [0–5 AU/mL]	--	--	73.08 (48.03; [59.14; 87.03]	99.85
25-hydroxyvitamin D [30–50 ng/dL]	33.05 (13.03; [30.84;35.26])	29.80	32.14 (15.39; [27.72;36.56])	28.30
Sodium [mmol/L]	139.72 (3.38; [139.29;140.14])	140	138.65 (3.17; [138.01;139.30])	139
Potassium [mmol/L]	4.48 (0.59; [4.40;4.56])	4.39	4.52 (0.63; [4.39;4.65])	4.48

This table shows biological parameters expressed as mean value (SD; CI: []) and median. The reference ranges for the biological parameters are presented according to standard clinical guidelines. For the biological parameters sodium, potassium, and 25-hydroxyvitamin D, the reference ranges are detailed by age and sex in Table 1. Abbreviations: SD—standard deviation; CI—confidence interval.

## Data Availability

Personal medical data are publicly unavailable due to privacy and ethical restrictions, having been obtained from the medical records of patients admitted to the Emergency Clinical Hospital for Children “Sf. Ioan”, Galati.

## Abbreviations

The following abbreviations are used in this manuscript:

ADH	Antidiuretic hormone
COVID-19	Coronavirus Disease 2019
CRP	C-reactive protein
EBV	Epstein–Barr virus
ESR	Erythrocyte sedimentation rate
EBNA	Epstein–Barr nuclear Antigen
IgM antibodies	Immunoglobulin M antibodies
IgG antibodies	Immunoglobulin G antibodies
IL-6	Interleukin-6
25-OH vitamin D	25-Hydroxyvitamin D
SARS-CoV-2	Severe Acute Respiratory Syndrome-Related Coronavirus 2
VCA	Viral Capsid Antigen
WHO	World Health Organization
